# Condition openness is associated with better mental health in individuals with an intersex/differences of sex development condition: structural equation modeling of European multicenter data

**DOI:** 10.1017/S0033291721004001

**Published:** 2023-04

**Authors:** Tim C. van de Grift

**Affiliations:** 1 Department of Plastic, Reconstructive and Hand Surgery, Amsterdam UMC (VUmc), Amsterdam, the Netherlands; 2 Department of Medical Psychology, Amsterdam UMC (VUmc), Amsterdam, the Netherlands; 3 Amsterdam Public Health Institute, Amsterdam, the Netherlands

**Keywords:** Anxiety, depression, disorders of sex development, openness, stigma

## Abstract

**Background:**

Openness on one's health condition or (stigmatized) identity generally improves mental health. Intersex or differences of sex development (DSD) conditions have long been kept concealed and high levels of (internalizing) mental health problems are reported. This study examines the effects of condition openness on anxiety and depression and the role of mediating concepts in this population.

**Methods:**

Cross-sectional data of individuals of 16 years and older with an intersex/DSD condition was collected in 14 specialized European clinics as part of the dsd-LIFE study. Patient-reported measures were taken on openness and shame (Coping with DSD), self-esteem (Rosenberg Self-Esteem Scale), satisfaction with care (CSQ4), anxiety and depression (HADS). Scores were compared per clinical group and data were analyzed via structural equation modeling (SEM) to calculate prediction and mediation models.

**Results:**

Data of 903 individuals were included in this study (Turner syndrome (*n* = 284), 46, XY DSD (*n* = 233), CAH (*n* = 206) and Klinefelter syndrome (*n* = 180)). Participants were moderately open on their condition. High levels of both anxiety and depression were observed across the sample. In SEM analysis, the tested models predicted 25% of openness, 31% of anxiety and 48% of depression. More condition openness directly predicted lower anxiety and depression symptoms, as well as indirectly through increased self-esteem, self-satisfaction and satisfaction with social support.

**Conclusions:**

Condition openness is associated with lower anxiety and depression in individuals with an intersex/DSD condition. Healthcare may provide the necessary knowledge and skills to employ one's optimal level of self-disclosure in order to improve mental health.

## Introduction

Differences of sex development (DSD or intersex conditions) encompass a group of variations of sex development, resulting in atypical sex chromosomes (XY in females, XXY in males etc.), gonads (testes in females, ovotestes etc.) and/or sexual anatomy (genital a-typicality or ambiguity etc.). Examples of intersex/DSD conditions include the Turner and Klinefelter syndrome (45, XO females, 47, XXY males), congenital adrenal hyperplasia (CAH: excessive androgen exposure and virilization in 46, XX females) and conditions with 46, XY karyotype (i.e. complete androgen insensitivity syndrome (CAIS) and complete gonadal dysgenesis with female assignment without virilization, partial androgen insensitivity syndrome (PAIS) and partial gonadal dysgenesis in both individuals assigned as males and females with partial virilization, and conditions such as severe hypospadias in assigned males). Depending on the definition, the prevalence of intersex/DSD has been reported to be up to 1/200 individuals (Lee et al., 2016). Yet, despite the substantial size of this group, individuals with intersex/DSD are seldom visible in society.

The societal invisibility of individuals with intersex/DSD is often associated with the decades in which the medical protocols based on the *gender neutrality theory* were applied to the counseling and treatments of this group (Fausto-Sterling, 2000). John Money published his gender neutrality theory in the 1970's in the light of the nature/nurture discussion of sex and gender (Money & Ehrhardt, 1972). Based on his theory that gender could be fully nurtured, clinical care of infants with intersex/DSD included early normalizing surgery (mostly feminizing), gender-typical upraising and no disclosure by professionals and parents to anyone, including the individuals themselves; in order to avoid gender ‘confusion’ (Fausto-Sterling, 2000). Not only has the gender neutrality theory been falsified (Diamond, 1982), follow-up studies have objectified that the clinical protocol of non-disclosure resulted in shame, stigma, dependency and trust issues toward parents and healthcare professionals and seemed to induce mental health problems (van Heesch, 2016). Also, early non-consensual ‘normalizing’ genital surgery is still frequently performed at present, and is thought to contribute to issues related to (medical) trauma, invisibility, decreased body image and mental health (Khanna, 2021; van Heesch, 2016).

Recent studies in adults with intersex/DSD show that mental health problems are generally more prevalent than in normative samples. This finding has been observed across the clinical conditions (Bruining, Swaab, Kas, & van Engeland, 2009; de Vries et al., 2019; Engberg et al., 2015; Schmidt et al., 2006; Schützmann, Brinkmann, Schacht, & Richter-Appelt, 2009). The levels of internalizing problems and suicidal ideation and attempts (6.7%) are specifically increased, suggesting a possible relationship with coping with the condition (de Vries et al., 2019). At the same time, the different underlying clinical conditions are generally characterized by varying (visible) physical intersex/DSD traits and levels of psychosocial impairment, possibly influencing the level of condition openness per clinical subgroup (Lee et al., 2016).

While present protocols advise full disclosure by health care providers on topics such as diagnosis, treatments and prognosis to individuals with intersex/DSD (Lee et al., 2016), many themselves still experience difficulties with self-disclosure or condition openness to others (Ernst et al., 2016). Population non-specific evidence from psycho-behavioral science shows that self-concealment (consciously concealing personal/negative information through avoidance or lying) is associated with more avoidant coping, and results in secret preoccupation, unfulfilled autonomy and increased isolation (Chaudoir & Fisher, 2010; Uysal, 2020). Furthermore, self-concealment is associated with increased anxiety, depression and suicidal behavior. On the other hand, self-disclosure (or openness) enables intimacy, receiving support, meaning-making and improved health outcomes (Uysal, 2020). Therefore, openness is thought to be amongst the key concepts for healthcare professionals to support individuals with stigmatized identities (Chaudoir & Fisher, 2010).

Health care providers can assist individuals by providing the required information about oneself and offering a safe space to practice self-disclosure. Therefore, contemporary scholars emphasize the importance of full disclosure from healthcare providers to individuals with intersex/DSD conditions (D'Alberton, 2010; Nordenström & Thyen, 2014). Full disclosure seeks to support the process of sense making and developing ownership (D'Alberton, 2010), and should take age (Nordenström & Thyen, 2014), culture (Weidler & Peterson, 2019) and the health care setting into consideration (Hertweck & Rothstein, 2019; Lundberg, Roen, Hirschberg, & Frisén, 2016). Still, many experience the information they received as too medicalized (Lundberg et al., 2016), which highlights the importance of the involvement of the mental health professional (Dessens et al., 2017).

Several studies described how individuals with intersex/DSD conditions cope with condition openness. Some recall a childhood of silence and uncertainty around their bodies and treatments, and experienced limited access to their medical data (MacKenzie, Huntington, & Gilmour, 2009). The limited societal knowledge on intersex/DSD is associated with embarrassment and limited disclosure by some (Carroll, Graff, Wicks, & Thomas, 2020). Adolescents that did disclose their condition to peers (with intersex/DSD) are mostly motivated by relationship trust and feeling a responsibility to disclose, as well as by lowering the burden of secrecy (Ernst et al., 2016). Barriers to disclosure include fears of rejection or being viewed as deviant, and not feeling skilled in what/how to disclose. Many prefer more healthcare support in acquiring these skills (Ernst et al., 2016).

Some factors have been associated with condition openness in individuals with intersex/DSD, such as self-esteem (van de Grift, Cohen-Kettenis, de Vries, & Kreukels, 2018). While direct associations between condition openness and mental health outcomes have been observed in other samples (Kosciw, Palmer, & Kull, 2015), these relationships have not been subject of study in this population. Given the long-term effects of concealment-based care on mental health in this group, and the effects of openness on mental health in other (stigmatized) samples, this study aims to objectify these relationships in a large cohort of individuals with intersex/DSD. Specific aims are (1) to assess the predictors of condition openness condition by individuals with intersex/DSD (2) to objectify direct/mediating pathways between openness and internalizing mental health problems (anxiety and depression), and lastly (3) to test the contribution of specific clinical DSD diagnoses to these relationships.

## Material and methods

### Procedure

Data were collected as part of the dsd-LIFE study, conducted in 14 specialized centers in France, Germany, the Netherlands, Poland, Sweden and the United Kingdom. The study sought to collect medical and patient-reported long-term outcome data on the wellbeing and quality of life of individuals across the intersex/DSD spectrum (Röhle et al., 2017). The study protocol was designed in collaboration with medical and mental health clinicians, support group representatives and ethicists. Ethical approval was received in all participating centers,^†^^1^ including the coordinating site (Charité Universitaetsmedizin, Berlin, Germany) and was registered in the German Clinical Trials Register (no. DRKS00006072).

Individuals were considered eligible to participate when being at least 16 years old and when having received any of the aforementioned intersex/DSD diagnoses. Candidates were approached through healthcare professionals in each participating site as well as via local support groups. Between February 2014 and September 2015 an approximate of 3100 candidates were approached, of whom 36% (*n* = 1040) consented to participate. Upon providing written informed consent, participants could participate in either completing the online patient-reported outcome questionnaires (minimum requirement), consent to retrieve medical data from patient files, and/or attend hospital visits for additional physical examinations. Upon request, participants could receive a paper version of the questionnaires or be assisted by independent research staff. Participants received several reminders by mail/telephone when they had not completed the minimum required data. For the purpose of this study, only questionnaire data and information from patient files will be used. After data collection was completed, diagnostic information was reviewed centrally on accuracy for all participants, and participants were allocated to diagnostic groups for analyses. A detailed protocol paper has been published earlier (Röhle et al., 2017).

### Theoretical model

**Fig. 1.**
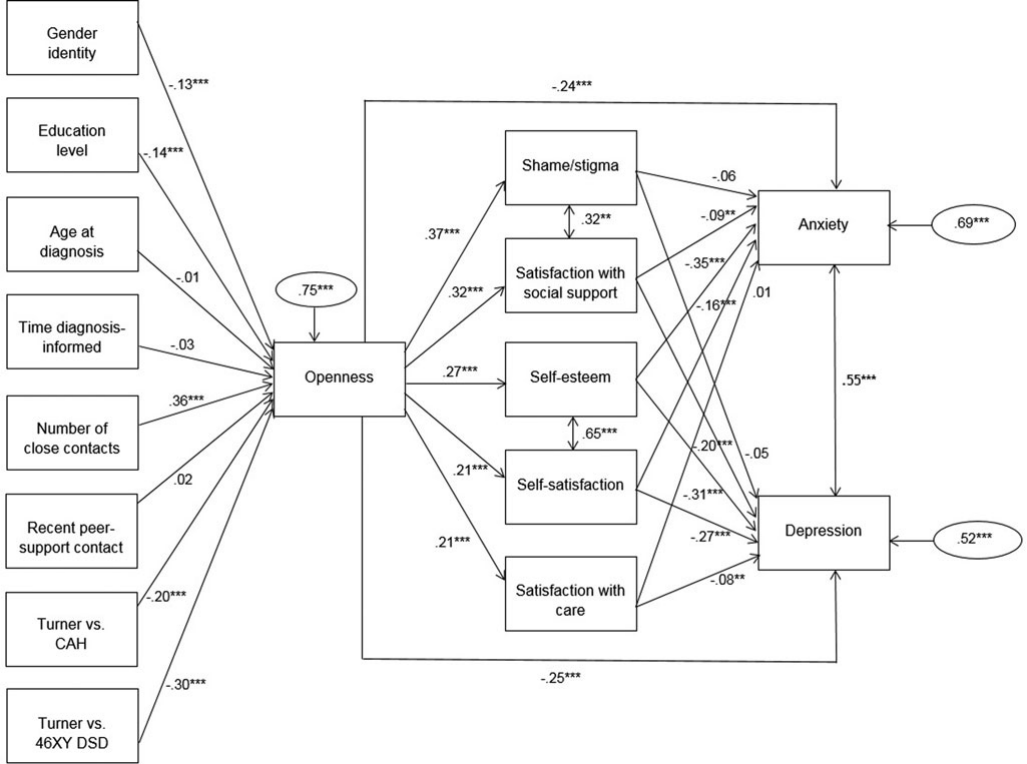
Structural equation model of predictors of openness, anxiety and depression and mediating factors (*n* = 775). * *p* < 0.05, ** *p* < 0.01, *** *p* < 0.001; Predictors of openness: male = 1, female = 0; recent peer-support contact: yes = 1, no = 0; dummy variables: Turner = 0, CAH/46,XY DSD = 1; Predictors of anxiety/depression: higher scores correspond with more openness, self-esteem, satisfaction and lower shame/stigma; Anxiety/depression: higher scores correspond with more symptoms CAH = Congenital Adrenal Hyperplasia; DSD = Disorders/differences of sex development.

Based on findings from intersex/DSD and non-intersex/DSD studies, a theoretical model with predictors of condition openness was constructed (Fig. 1). Predictors included demographic, socio-economic, clinical, and psychological factors. The following predictors of self-disclosure or condition-openness were derived from the literature or added to the model based on intersex/DSD-specific hypotheses (populations between brackets): gender [individuals with psychiatric conditions (Yokoyama et al., 2019)], educational level [individuals with psychiatric conditions (Husky, Zablith, Fernandez, & Kovess-Masfety, 2016)], age [individuals with psychiatric conditions (Husky et al., 2016)], age at diagnosis and time between being diagnosed and informed (based on clinical hypothesis), social and peer-support contacts [individuals with psychiatric conditions (Husky et al., 2016)] and psychological counseling received [sexual minorities (Ali & Barden, 2015)]. Furthermore, the following mediators between self-disclosure/condition openness and mental health outcomes were derived from the literature (populations between brackets): satisfaction with social support [individuals with HIV (Niu et al., 2019)], shame/stigma [individuals with psychiatric conditions (Yokoyama et al., 2019)], self-esteem [individuals with psychiatric conditions (Yokoyama et al., 2019)], satisfaction with self [individuals experiencing distress (Kahn, Wei, Su, Han, & Strojewska, 2017)], and satisfaction with care [lesbian women (Polek, Hardie, & Crowley, 2008)]. Direct relationships between openness and anxiety/depression have been observed in populations with HIV (Niu et al., 2019) and with male infertility (Babore, Stuppia, Trumello, Candelori, & Antonucci, 2017).

### Participants

Due to the sampling strategy, no background characteristics comparisons could be made between the participating and non-participating groups. Because of the statistical requirements, only participants with data available on all the outcome variables and with a binary gender were included for analyses, resulting in a final sample of 903 participants (87%). Compared with the excluded sample, included participants were higher educated (Cramer's *V* = 0.15) and reported somewhat more close contacts (Cramer's *V* = 0.12) on average. Also, more participants with Klinefelter syndrome were excluded. No differences were observed in the other variables (Table 1).

**Table 1.** Sample characteristics, *n* = 1040

	Included participants *n* = 903 (87%)	Excluded participants *n* = 137 (13%)	Test statistics
Birth-assigned sex			χ^2^(11 040) = 12.6, *p* < 0.001, Cramer's *V* = 0.11
Female	649 (72)	78 (57)	
Male	254 (28)	59 (43)	
Gender identity, *n* (%)			χ^2^(41 040) = 90.6, *p* < 0.001, Cramer's *V* = 0.30
Female	646 (72)	71 (52)	
Male	257 (29)	39 (39)	
Open (i.e. non-binary)	0 (–)	2 (2)	
Inter(sex)	0 (–)	7 (5)	
Other	0 (–)	3 (2)	
Educational level, *n* (%)			χ^2^(2921) = 19.7, *p* < 0.001, Cramer's *V* = 0.15
Lower	172 (20)	30 (39)	
Intermediate	415 (49)	38 (49)	
Higher	258 (31)	9 (12)	
Age at participation, years, M (s.d.)	32.2 (13.0)	33.6 (17.1)	*t*(1038) = 1.1, *p* = 0.27, Cohen's *d* = 0.09
Age at diagnosis, years, Mdn (IQR)	9 (0–16)	11 (0-18)	*U* = 43 633, *p* = 0.10, Cohen's *d* = 0.11 χ^2^(4955) = 6.1, *p* = 0.20, Cramer's *V* = 0.08
Before/at birth, *n* (%)	261 (30)	16 (20)	
Infancy (<3 years)	95 (11)	7 (9)	
Childhood (4–12 years)	169 (19)	14 (18)	
Adolescence (13–17 years)	175 (20)	19 (24)	
Adulthood (⩾18 years)	176 (20)	23 (29)	
Diagnosis, *n* (%)			χ^2^(51 040) = 16.0, *p* = 0.007, Cramer's *V* = 0.12
Turner syndrome^a^	284 (32)	41 (30)	
Congenital Adrenal Hyperplasia^§^	206 (23)	20 (15)	
46,XY DSD			
Non-virilized females	101 (11)	9 (7)	
Partial-virilized females	58 (6)	8 (6)	
Males	74 (8)	14 (10)	
Klinefelter syndrome^a^	180 (20)	45 (33)^b^	
Number of close contacts, *n* (%)			χ^2^(6980) = 13.1, *p* = 0.04, Cramer's *V* = 0.12
None	53 (6)	8 (10)	
1	108 (12)	13 (16)	
2	171 (19)	23 (28)	
3	220 (25)	19 (24)	
4–6	256 (29)	13 (16)^b^	
7–9	50 (6)	1 (1)	
⩾10	41 (5)	4 (5)	
Relationship status			χ^2^(3990) = 4.4, *p* = 0.22, Cramer's *V* = 0.07
Having a partner	346 (38)	36 (41)	
Living alone	231 (26)	15 (17)	
Living with parents	290 (32)	31 (35)	
Other/not applicable	35 (4)	6 (7)	
Sexual orientation			χ^2^(3947) = 11.0, *p* = 0.01, Cramer's *V* = 0.11
(primarily) attracted to men	502 (57)	21 (36)^b^	
(primarily) attracted to men/women	31 (4)	2 (3)	
(primarily) attracted to women	263 (30)	23 (40)	
other	93 (11)	12 (21)^b^	
Recent peer-support contact, *n* (%)			χ^2^(1974) = 0.27, *p* = 0.76, Cramer's *V* = 0.02
Yes	160 (18)	13 (16)	
No	731 (82)	70 (84)	
Lifetime psychological counseling, *n* (%)			
Any	341 (39)	25 (33)	χ^2^(1963) = 1.1, *p* = 0.33, Cramer's *V* = 0.03 χ^2^(1910) = 0.87, *p* = 0.39, Cramer's *V* = 0.03 χ^2^(1926) = 0.47, *p* = 0.48, Cramer's *V* = 0.02
Childhood or adolescence	204 (24)	14 (19)	
Adult	244 (28)	21 (32)	

DSD, Disorders/differences of sex development.

a Including genetic mosaics; ^§^including simple virilizing, salt-wasting subtypes.

b Standardized Z-score significantly different in posthoc testing.

### Measures

The following measure was collected as study outcome:

– **Hospital Anxiety and Depression Scale (HADS-A/D)**: This 14-item self-report scale surveys experienced anxiety and depressive symptoms over the past week (Zigmond & Snaith, 1983). The anxiety (HADS-A) and depression (HADS-D) subscale correspond with the DSM-IV and ICD-10 clinical diagnoses. Participants rate their agreement with statements (e.g. ‘I feel tense and wound up’) on 4-point Likert scales (e.g. from ‘most of the time’ to ‘not at all’). Reliability of the scales was good for HADS-A (Crohnbach's *α* = 0.81) and acceptable for HADS-D (Crohnbach's *α* = 0.78), which is comparable to values in other samples. HADS-A/D subscale sum scores were used as outcome variables.

The following measures were collected as study predictors and mediators:

– **Coping with DSD Scale**: In this scale participants rate their agreement with nine statements on a 4-point Likert scale (from ‘completely true’ to ‘not true at all’) (Kleinemeier et al., 2010). The measure yields two subscales: the openness scale (five items; e.g. ‘I can talk openly to my friends about my condition’) and the shame/stigma scale (four items; e.g. ‘My body embarrasses me’). Higher scores correspond with more openness (possible range = 5–20) and less shame/stigma (possible range = 4–16). Due to possible construct overlap, all items from the Coping with DSD scale and Rosenberg Self-Esteem Scale were analyzed together in confirmatory factor analysis. The results confirmed the three factors included in the two measures (normalized χ^2^(149–903) = 7.4, *p* < 0.001, CFI = 0.9, TLI = 0.9, RMSEA = 0.08, SRMS = 0.07) and showed that all but one of the original Coping with DSD scale items contributed significantly to these three factors. This item (‘Afraid to tell sexual partner about my condition’) was found to perform poorly before (Kleinemeier et al., 2010) and was replaced by a similar yet contributing newly added item in this study (‘I can talk freely about my condition’). As a result, the scale reliability was acceptable for both the openness (Crohnbach's *α* = 0.76) and the shame/stigma scale (Crohnbach's *α* = 0.75). No earlier measure psychometric characteristics have been reported to compare.
– **Rosenberg Self-Esteem Scale (RSES)**: This scale assesses the level of trait-like global self-esteem through the level of agreement with 10 statements (Rosenberg, 1965). Statements were rated on a 4-point Likert scale from ‘strongly disagree’ to ‘strongly agree’ (e.g. ‘I take a positive attitude toward myself’). Higher scores represent a higher self-esteem. The RSES showed excellent reliability in the present sample (Crohnbach's *α* = 0.91).
– **Customer Satisfaction Scale (CSQ-4)**: In this four-item scale, participants rate their satisfaction with services they receive(d) on 4-point Likert scales ranging from negative to positive (Attkisson & Greenfield, 1995). The items survey whether the services met one's needs, whether one would choose them again, their general satisfaction and whether they helped in dealing with one's condition. Higher sum scores refer to higher satisfaction with care. The CSQ4 yielded good reliability in the present sample (Crohnbach's *α* = 0.80).
– **Quality of life**: Two items were used from the WHO quality of life scale. On self-satisfaction: ‘How satisfied are you with yourself?’ and on satisfaction with support ‘How satisfied are you with the support you get from your friends?’ (Whoqol Group, 1998). Participants rated their level of satisfaction over the past two weeks on a 5-point Likert scale (from ‘very dissatisfied’ to ‘very satisfied’).

The following background information was collected from self-report measures or retrieved from patient files more info in Röhle et al., 2017):

– **Sociodemographic data**: gender identity (male, female, open (i.e. non-binary), inter, third, other), education level (conform the European Social Survey: lower = lower secondary education or less, intermediate = upper secondary or vocational education, higher = tertiary education or higher), age at participation (open), age at diagnosis and when being informed (multiple choice), number of close contacts (multiple choice), living situation (with partner, alone, with parents, other), sexual orientation (primarily to men, women, both, other), recent peer-support contact (yes/no), whether participants had received psychological support during childhood, adolescence and adulthood (yes/no), and the clinical diagnosis.
– **Clinical data**: information on the clinical diagnosis was retrieved from patient records and combined with patient-reported diagnosis into diagnostic classes according to the DSD consensus statement (Lee et al., 2016; Röhle et al., 2017).

### Analyses

Participants were allocated over six diagnostic subgroups based on clinical characteristics (Turner syndrome, CAH, 46,XY DSD female without androgen effects, 46, XY female with partial androgen effects, 46, XY male, and Klinefelter syndrome) and earlier studies (Kleinemeier et al., 2010; Röhle et al., 2017). The questionnaire data were recoded and scored according to the available manuals. Variables were recoded for education, any psychosocial support and diagnostic classes. For the openness variable, data were imputed (based on scale mean) when participants did not miss more than 20% of the scale variables. Otherwise, participants were excluded from analysis. A sensitivity analysis of the imputed data was performed by correlating both the original and imputed variable to the HADS subscales for comparison. Data dummies were generated for the diagnostic categories, using the Turner sample as reference group (being the largest). Included and excluded participants were compared on background characteristics using independent sample *t* test (continuous variables) and chi-squared tests (nominal and ordinal variables). Cohen's *d*, Cramer's V and Eta squared (*η*^2^) values were calculated as effect size measures as appropriate. Mean scores of the openness, mediating concepts and anxiety and depression measures were tested for statistically significant differences between the diagnostic subgroups using one-way ANOVA and chi-squared tests. Posthoc testing was performed applying Bonferroni testing or standardized Z-scores. All hypothesized predictors, mediators and outcomes (see *Theoretical model*) were associated via simple correlations. Cross-country comparisons were not part of the present study protocol. Based on the study aim and suggestions for purposeful variable selection (Heinze, Wallisch, & Dunkler, 2018), variables with a correlation of *p* ⩽ 0.10 with openness were put forward into the structural equation modeling (SEM). The SEM model tested three regression analyses: one analysis predicting openness, one predicting anxiety and one predicting depression. The regressions on anxiety and depression included both direct effects of the factors as well as interaction effects of the factors with openness (calculated by multiplying standardized Z-scores). Interaction effects were tested hypothesizing that some mediators had conceptual overlap and/or may be subject to underlying psychological traits. Additionally, residual variance was calculated for the three outcome variables. Statistical testing of the scale reliability, the sample descriptives, subgroup differences and correlations were conducted using IBM SPSS statistics 26.0. The CFA (performed for the Coping with DSD scale only) and SEM analyses were performed in R studio, using the *lavaan* package (Rosseel, 2012).

## Results

### Sample characteristics

Sensitivity analysis showed no significant differences between the correlations of the non-imputed and imputed openness variable to the HADS subscales (anxiety: *r* = −0.244 *v. r* = −0.236 and depression: *r* = −0.257 *v. r* = −0.264). The participants included for analyses (*n* = 903; Table 1) were mostly moderate to higher educated, a majority identified as female and with a mean age of 32 years. Eighty percent of participants received their clinical diagnosis during childhood or adolescence. The largest clinical samples included participants with Turner syndrome (*n* = 284) and with 46, XY DSD (*n* = 233) followed by those with CAH (*n* = 206) and Klinefelter syndrome (*n* = 180). The majority reported having at least three close contacts and a minority had been in contact with peers (with intersex/DSD) over the past year. Around two-fifth had received (any) psychological counseling during their lives.

### Levels of openness, anxiety and depression

The levels of openness, anxiety and depression, as well as the scores of the mediating variables are reported per clinical group in Table 2. Participants were moderately open on their condition, with participants with Turner syndrome being most open, and males with a 46, XY DSD being least open (*η*^2^ = 0.12). On average participants reported they experienced somewhat shame and/or stigma regarding their intersex/DSD condition, without subgroup differences. The mean HADS-A scores ranged from 6.7 (46, XY males) to 8.1 (46, XY DSD partly virilized females) without significant subgroup differences, and the mean HADS-D scores ranged from 3.6 (Turner syndrome) to 5.5 (Klinefelter syndrome). The latter group scored significantly higher than most other subgroups (*η*^2^ = 0.04). Self-esteem was moderately positive on average with little between-group differences. Satisfaction with care was fairly high on average, with participants with Turner syndrome and CAH being most satisfied, and those with Klinefelter syndrome being least satisfied. Around 60–80% was (very) satisfied with the support they received, whereas slightly lower percentages were reported on satisfaction with oneself.

**Table 2.** Descriptive characteristics of the mediating and outcome variables, *n* = 903

		46, XY DSD					
	1 Turner syndrome *n* = 284 (32%)	2 Congenital adrenal hyper-plasia *n* = 206 (23%)	3 Non-virilized females *n* = 101 (11%)	4 Partially viri-lized females *n* = 58 (6%)	5 Males *n* = 74 (8%)	6 Klinefelter syndrome *n* = 180 (20%)	Test statistics
DSD openness scale (coping with DSD scale), M (s.d.)^a^	14.7 (3.3)	12.7 (3.9)	12.6 (4.0)	10.8 (3.2)	10.6 (3.3)	13.4 (4.0)	*F*(5897) = 24.0, *p* < 0.001, *η*^2^ = 0.12 1 > all 2,3 and 6 > 4 and 5
DSD shame/stigma scale (coping with DSD scale), M (s.d.)^b^	12.2 (2.8)	12.2 (3.1)	12.2 (3.2)	11.8 (3.1)	11.0 (3.5)	11.9 (3.3)	*F*(5897) = 1.9, *p* = 0.08, *η*^2^ = 0.01
Self-esteem (RSES), M (s.d.)^c^	19.0 (5.6)	20.8 (6.1)	20.6 (6.4)	20.3 (5.7)	19.5 (6.2)	19.5 (6.2)	*F*(5897) = 2.7, *p* = 0.02, *η*^2^ = 0.02 2 > 1
Satisfaction with care (CSQ4), M (s.d.)^d^	13.6 (1.9)	13.4 (2.3)	12.7 (2.5)	12.7 (2.1)	12.8 (2.2)	12.7 (2.5)	*F*(5897) = 6.0, *p* < 0.001, *η*^2^ = 0.03 1 > 3 and 6 2 > 6
Anxiety (HADS-A), M (s.d.)^e^	6.8 (4.0)	6.9 (4.0)	7.0 (4.5)	8.1 (4.1)	6.7 (3.8)	6.8 (4.2)	*F*(5897) = 1.2, *p* = 0.29, *η*^2^ = 0.01
Depression (HADS-D), M (s.d.)^e^	3.6 (2.8)	4.3 (3.6)	4.1 (3.9)	4.1 (3.2)	3.9 (3.4)	5.5 (3.9)	*F*(5897) = 7.0, *p* < 0.001, *η*^2^ = 0.04 6 > 1,2,3 and 5
Satisfaction with support (WHO Qol-bref), *n* (%) Very dissatisfied Dissatisfied Neither/nor Satisfied Very satisfied	1 (0)^f^ 14 (5) 50 (18) 146 (51) 73 (26)	4 (2) 15 (7) 48 (23) 74 (36) 65 (32)^f^	3 (3) 4 (4) 23 (23) 45 (45) 26 (26)	0 (-) 5 (9) 16 (28) 24 (41) 13 (22)	2 (3) 8 (11) 17 (23) 38 (51) 9 (12)^f^	10 (6)^f^ 15 (8) 51 (28) 73 (41) 31 (17)^f^	χ^2^(20 903) = 48.2, *p* < 0.001, Cramer's *V* = 0.12
Self-satisfaction (WHO Qol-bref), *n* (%) Very dissatisfied Dissatisfied Neither/nor Satisfied Very satisfied	6 (2) 30 (11) 95 (34) 124 (44) 29 (10)	5 (2) 21 (10) 55 (27) 93 (45) 32 (16)	6 (6) 7 (7) 22 (22) 52 (52) 14 (14)	1 (2) 4 (7) 16 (28) 29 (50) 8 (14)	2 (3) 7 (10) 21 (28) 30 (41) 14 (19)	5 (3) 22 (12) 41 (23) 82 (46) 30 (17)	χ^2^(20 903) = 20.4, *p* = 0.44, Cramer's *V* = 0.08

DSD, Disorders/differences of sex development.

a Score ranges from 5 (least open) to 20 (most open).

b Score ranges from 4 (most stigma/shame) to 16 (least stigma/shame).

c Score ranges from 0 (lowest self-esteem) to 30 (highest self-esteem).

d Score ranges from 4 (lowest satisfaction) to 16 (highest satisfaction).

e Score ranges from 0 (least symptoms) to 20 (most symptoms).

f Standardized Z-score significantly different in posthoc testing.

### SEM outcomes – openness

Based on the correlation analyses, the following variables did not reach the threshold of *p* ⩽ 0.10 (in relation to openness by individuals with intersex/DSD) and were therefore dropped from the model: age at participation, living situation, sexual orientation, dummy variable of Turner *v.* Klinefelter, and lifetime psychological counseling (remaining variables associated in Table 3). The final model, including participants without missing data on any of the measures (*n* = 775, missings mostly included background variables) performed acceptable (Fig. 1; normalized χ^2^(26 775) = 8.0, *p* < 0.001, CFI = 0.9, TLI = 0.7, RMSEA = 0.09, SRMS = 0.04). A total of 25% of variance in openness by individuals with intersex/DSD was explained by the included variables. Significant predictors of more condition openness included having more close contacts (*β* = 0.36), belonging to the Turner syndrome group (*β* = −0.30 and *β* = −0.20; dummy variables: Turner = 0, CAH/46, XY DSD = 1), a lower education (*β* = −0.14) and being female (*β* = −0.13; female = 0, male = 1), whereas age at diagnosis, time between diagnosis and being informed and recent peer-support contact did not contribute significantly.

**Table 3.** Correlations between predicting, mediating and outcome variables (*n* = 903), *r*

	1.	2.	3.	4.	5.	6.	7a.	7b.	8.	9.	10.	11.	12.	13.	14.	15.
1. Gender (binary)^a^*	–	−0.17***	0.25***	0.02	−0.14***	−0.05	−0.32***	0.04	−0.11**	−0.16***	−0.09**	−0.03	0.02	−0.12***	−0.02	0.14***
2. Education level		–	0.01	0.05	0.18***	0.09**	−0.02	0.06*	−0.07*	0.05	0.04	0.13***	0.02	0.00	−0.07*	−0.12***
3. Age at diagnosis (categories)			–	−0.18***	0.06*	0.09*	−0.36***	−0.15***	0.09**	−0.098	−0.01	−0.07*	−0.10**	−0.09**	0.03	0.13***
4. Time diagnosis – informed				–	−0.11**	−0.01	−0.02	0.15***	−0.12***	−0.14***	−0.12***	−0.09**	−0.10**	−0.09**	0.08*	0.13***
5. No. of close contacts					–	0.12***	−0.08*	0.02	0.35***	0.39***	0.23***	0.26***	0.17***	0.18***	−0.20***	−0.31***
6. Recent peer-support contact^b^						–	−0.07*	−0.05	0.09**	0.01	−0.06*	−0.07*	−0.02	−0.10**	0.00	0.07*
7a. Turner *v.* CAH^c^							–	−0.32***	−0.07*	0.04	0.03	0.09**	0.02	0.06*	−0.01	0.01
7b. Turner *v.* 46, XY DSD^c^								–	−0.25***	−0.04	−0.05	0.04	0.03	−0.11**	0.04	−0.04
8. Openness^d^									–	0.32***	0.37***	0.27***	0.20***	0.21***	−0.24***	−0.26***
9. Satisfaction with support^d^										–	0.32***	0.39***	0.38***	0.34***	−0.32***	−0.47***
10. Shame/stigma^d^											–	0.47***	0.47***	0.29***	−0.35***	−0.41***
11. Self-esteem^d^												–	0.65***	0.26***	−0.53***	−0.61***
12. Satisfaction with self^d^													–	0.29***	−0.46***	−0.59***
13. Satisfaction with care^d^														–	−0.19***	−0.33***
14. Anxiety^e^															–	0.55***
15. Depression^e^																–

CAH, congenital adrenal hyperplasia; DSD, disorders/differences of sex development.

*p < 0.10, ** *p* < 0.01, *** *p* < 0.001.

^a^ Male = 1, female = 0.

b Yes = 1, no = 0.

c Dummy variables: Turner = 0, CAH/46XY DSD = 1.

d Higher scores correspond with more openness, self-esteem, satisfaction and lower shame/stigma.

e Higher scores correspond with more symptoms.

### SEM outcomes – anxiety and depression

The HADS-A and HADS-D scores were moderately positively associated, comparable to values from the literature. The level of openness significantly predicted all mediating factors; with the strongest relationship with shame/stigma (*β* = 0.37). The level of anxiety symptoms was predicted for 31% by the model, while the level of depressive symptoms was predicted for 48% by the model. Higher levels of anxiety were predicted by less openness (*β* = −0.24) directly, as well as by three moderating concepts: lower self-esteem (*β* = −0.35), lower self-satisfaction (*β* = −0.16) and lower satisfaction with social support (*β* = −0.09). The levels of shame/stigma experienced and satisfaction with care did not contribute significantly independently. The strongest interactional effect influencing the level of anxiety was openness × self-satisfaction (*β* = 0.06). Higher levels of depression were predicted by less openness (*β* = −0.25) directly, as well as by four mediating concepts: lower self-esteem (*β* = −0.31), lower self-satisfaction (*β* = −0.27), lower satisfaction with social support (*β* = −0.20) and lower satisfaction with care (*β* = −0.08). Similarly as reported for anxiety, the level of shame/stigma experienced did not contribute significantly to the model. Furthermore, the strongest interactional effect contributing to depressive symptoms was openness × self-satisfaction (*β* = 0.10).

## Discussion

Openness about having an intersex/DSD condition by individuals experiencing it has long been discouraged to individuals (and families) as it was thought to be harmful to mental health. The increasing insight in the negative consequences of concealment-based intersex/DSD care (van Heesch, 2016), and knowledge on how self-disclosure supports mental health outcomes in other populations (Uysal, 2020) has led to openness-supporting healthcare. This study is the first to quantify the relationship between condition openness and mental health outcomes in a large cross-condition sample of adults with an intersex/DSD condition.

In general, participants were moderately open on their condition to parents, friends and others. Moreover, the Coping with DSD scale values' standard deviations point out the large variability within the sample, whereas this differed somewhat per clinical subgroup. These findings align with the literature describing that to date, individuals with intersex/DSD still face many difficulties with being open on their condition (Carroll et al., 2020; Ernst et al., 2016). For some, this may be the direct result of the concealment-based healthcare and/or non-consensual early normalizing genital surgery they receive(d), or having been discouraging to be open and of having insufficient knowledge and skills to discuss their bodies. For others, the limited openness may be rooted in the poor visibility of individuals with sex variations in society (Ernst et al., 2016). In the absence of role models and general knowledge on sex variations, people may fear misunderstanding, bullying or discrimination when being open. Contemporary studies show that individuals with intersex/DSD prefer more (practical) support in developing an appropriate story and skills to self-disclose and that this support should be tailored to specific phases of life (e.g. with first sexual partners) (Callens, Kreukels, & van de Grift, 2021; Carroll et al., 2020).

Some between-condition differences were observed in this study. Women with Turner syndrome reported the highest condition openness. This has not been reported before and appears to be in contrast with the stereotype of the shy nature of women with Turner syndrome (Schmidt et al., 2006). However, Turner syndrome did not always fall logically under the intersex/DSD umbrella and may have been less subject to the concealment-based healthcare, resulting in more openness. Also, the 45, XO karyotype may be perceived less as being ‘between the sexes’ and therefore less threatening to disclose. Lastly, Turner syndrome is often characterized by syndrome-specific socially visible physical built such as short stature and low-set ears, which can lead to more frequent self-disclosure. Least openness was reported by participants with 46, XY DSD, specifically males and females with partial virilization. These unfavorable scores have been reported earlier for German adolescents, although not statistically significant [likely due to insufficient power (Kleinemeier et al., 2010)]. Possibly, with having the highest degree of physical sex-ambiguity and the ambiguity being related to genitals specifically, these participants include the most stigmatized groups within the intersex/DSD spectrum. This seems to be supported by the most unfavorable shame/stigma scores in these groups as well. The mechanism of stigma attached to the sex ambiguity, and the negative impact on many areas of life, has been described for other intersex/DSD conditions (Carroll et al., 2020), but no cross-condition comparisons have been published before.

Factors other than the clinical diagnosis that significantly influenced the level of openness included female gender, lower education level and higher number of close contacts. More openness in females has been reported in other samples before (Yokoyama et al., 2019) and may relate to gender differences in general openness, in social acceptance of sex variation and in self-disclosure, all favoring females. The higher level of openness in lower-educated participants was unexpected and is in contrast with the literature (Husky et al., 2016). This finding appeared to be confounded by the lower age of the lower-educated participants that had not finished their final degrees. Since age at participation did not make it into the final model, the education level seems to include the effect of lower age on more openness as well. Possibly, individuals with higher education level may have strived for high education and job security as coping mechanism for internalized stigma, although this remains speculative. Such behavior has however been hypothesized for gay men earlier (Downs, 2012). Interestingly, having a higher number of close contacts also predicted a higher degree of condition openness, something that has been reported for people with psychiatric problems before (Husky et al., 2016). This relationship could imply that having more contacts provides an ability to practice openness and share bits of one's story with multiple people. Whereas it could also indicate the level of social and communicative skills one has. Interestingly, recent peer support contact did not predict the level of openness, possibly suggesting that openness within peer-groups may not necessarily generalize toward the rest of life. Also, having received psychological counseling did not correlate with condition openness: possibly due to the indications, as well as the differences in the scope and quality of psychological counseling. This relationship could be a subject of further study.

The mean anxiety and depression (HADS) scores for most clinical groups in this study were elevated compared with normative general population samples, and comparable to general medical patient samples or even elevated (Spinhoven et al., 1997). Again, the high standard deviation indicates the large variability in experienced symptoms within this group. The elevated levels of anxiety and depression have been reported in earlier studies on individuals with intersex/DSD (Chadwick, Smyth, & Liao, 2014; de Vries et al., 2019; Reisch et al., 2011). Possible factors that may contribute to these elevated levels include sex hormonal imbalances (and associated physical symptoms such as fatigue; Spinelli, 2004), lowered body image/self-esteem (van de Grift et al., 2018), and lower social acceptance and support (Kessler, Price, & Wortman, 1985).

The present study shed light on the role condition openness by individuals with intersex/DSD specifically plays in developing anxiety and depressive symptoms in this population. The tested model accounted for a substantial part of both the levels of anxiety and depression. More condition openness predicted less anxiety and depression symptomatology directly as well as through multiple mediators. These mediators included improvements in self-satisfaction and self-esteem, and an increased satisfaction with social support and with care (for depression only) as a result of more condition openness. These findings confirm theories on non-intersex/DSD samples, describing how condition openness facilitates meaning-making, intimacy and support (Chaudoir & Fisher, 2010; Uysal, 2020). Contrastingly, concealment can induce avoidant coping, secret preoccupation and unfulfilled autonomy (Uysal, 2020), which may all contribute to anxiety and depressive symptoms. The concepts mediating between openness and mental health in our study align with earlier findings on individuals with HIV, with psychiatric conditions, and on lesbian women (Niu et al., 2019; Polek et al., 2008; Yokoyama et al., 2019). More condition openness by individuals with intersex/DSD helps to inform others on one's needs and likely results in more suitable social support and healthcare. Also, by being open, one can experience affirmative encounters and build resilience toward negative responses. This again supports positive self-evaluation and feelings of accomplishment/agency. On the other hand, a lack of openness can lead to feelings of not being fully seen by others or having a less-integrated identity, all possibly contributing to lower self-evaluation and symptoms of anxiety (e.g. fear of others finding out) or depression (e.g. grieve over loss/isolation). The strongest interaction effect was observed between condition openness and self-satisfaction, which might protect against anxiety or depressive feelings through more positive cognitions about oneself in relation to others and affirmative social behavior for example.

### Limitations

The present study was subject to some methodological and conceptual limitations. Firstly, the cross-sectional design did not allow for definite conclusions on the direction of the relationships between openness, anxiety/depression and the mediators. Reverse causations cannot be ruled out: e.g. feelings of anxiety/depression can result in lower self-esteem, less support and less openness. However, (the direction of) our findings do align with the available literature. Other limitations include the limited psychometric validation of the Coping with DSD scale, the low participation rate, the heterogeneity of the sample and higher share of missing data in certain subgroups (e.g. the Klinefelter participants may have had trouble completing the survey and/or being less open about the studied subject). Future studies may replicate the present findings in each intersex/DSD condition specifically, especially in those identifying as intersex or non-binary. Also, study participants may have been more likely to be open about their intersex/DSD condition (i.e. higher educated, more close contacts), possibly reducing the study's generalizability. Furthermore, the concept of openness/self-disclosure has not been well-explored in individuals with intersex/DSD. While we surveyed a rather generic concept of openness, it is likely to be multifaceted in terms of where, to whom and what to disclose in each phase of life. This is also reflected in the substantial residual variance we observed for the different outcomes. Neither did we study openness of relatives and healthcare providers. Also, significant interactional effects were observed in the model, leaving contributing latent factors unexplored. More hypothesis-generating research, for example on the role of healthcare providers' openness, is therefore appropriate in this field.

## Conclusions

Despite the many years of concealment, at present, a significant number of individuals with intersex/DSD conditions reports some degree of openness on their variations of sex. Condition openness by individuals with intersex/DSD was found to be associated with positive mental health outcomes, both directly as well as through improved self-evaluation and satisfaction with support and care. The optimal degree of openness likely differs on a personal basis and remains unstudied, yet, findings in other populations suggest that openness can assist people in feeling more integrated and being seen for who they are, as well as connecting more to others. Providing a safe space for the understanding of oneself, one's story and practicing openness in each phase of life may therefore be part of healthcare for this group. Experienced patient, parent and healthcare providers' openness can be subject of further (qualitative) study in the future.

## The dsd-LIFE group are

Birgit Kohler and Uta Neumann, Berlin; Peggy Cohen-Kettenis, Baudewijntje Kreukels and Annelou de Vries, Amsterdam; Wiebke Arlt, Birmingham; Claudia Wiesemann, Gottingen; Jolanta Slowikowska-Hilczer, Lodz; Ute Thyen and Marion Rapp, Lubeck; Aude Brac de la Perriere, Lyon; Charles Sultan and Francoise Paris, Montpellier; Nicole Reisch, Munich; Annette Richter-Unruh, Munster and Bochum; Hedi Claahsen – van der Grinten, Nijmegen; Claire Bouvattier and Lise Duranteau, Paris; Anna Nordenström and Agneta Nordenskjöld, Stockholm; Catherine Pienkowski, Toulouse; Maria Szarras-Czapnik, Warsaw.

## Footnotes

† The notes appear after the main text.

## Footnotes

1 Institutions that issued ethical approval were as follows: In France: Université Claude Bernard Lyon; Le Centre Hospitalier Universitaire Montpellier; Assistance Publique – Hôpitaux de Paris; Le Centre Hospitalier Universitaire de Toulouse. In Germany: Charité Universitaetsmedizin Berlin; Ludwig-Maximilians-Universitaet, Munich; University of Luebeck; Universitaetsmedizin Goettingen; Westfaelische Wilhelms- Universitaet Muenster. In Poland: Medical University of Lodz; Children' Memorial Health Institute, Warsaw. Sweden: Karolinska Institutet, Stockholm. In The United Kingdom: University of Birmingham. In the Netherlands: VU University Medical Center, Amsterdam; Radboud University Nijmegen Medical Center, Nijmegen.
